# Characterization of the Polyphenolic Profile in Tomato (*Lycopersicon esculentum* P. Mill) Peel and Seeds by LC-HRMS/MS

**DOI:** 10.1021/acs.jafc.4c02126

**Published:** 2024-07-08

**Authors:** Jared Mauricio López-Téllez, María Del Pilar Cañizares-Macías, Aina Mir, Javier Saurina, Oscar Núñez

**Affiliations:** † Department of Analytical Chemistry, Faculty of Chemistry, Universidad Nacional Autónoma de México, Mexico City 04510, Mexico; ‡ Department of Chemical Engineering and Analytical Chemistry, Universitat de Barcelona, Barcelona E08028, Spain; § Research Institute in Food Nutrition and Food Safety, Universitat de Barcelona, Santa Coloma de Gramenet E08921, Spain; ∥ Serra Húnter Fellow Programme, Barcelona E08003, Spain

**Keywords:** phenolic compounds, *Lycopersicon esculentum* P. Mill, tomato byproducts, LC-HRMS, chemometrics

## Abstract

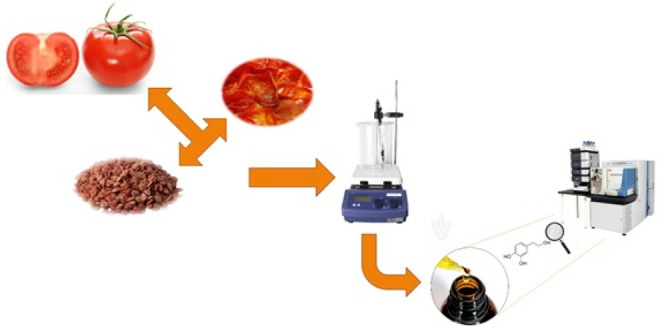

Peel and seeds are the main byproducts from tomato (*Lycopersicon esculentum* P. Mill) processing with high concentrations of polyphenols that have been underexploited. Herein, polyphenolic profiles in tomato peel and seeds were elucidated by untargeted liquid chromatography coupled to high-resolution mass spectrometry (LC-HRMS) with an LTQ Orbitrap analyzer. Samples from two Spanish regions—“Murcia” and “Almería”—were analyzed to obtain complementary results. 57 compounds were found, mainly phenolic acids and flavonoids, of which eight were identified for the first time in tomato. Polyphenols were more abundant in byproducts from “Murcia” samples than in those from“Almería” samples, where the abundance of compounds like coutaric, caffeic, neochlorogenic, dicaffeoylquinic and ferulic acids, vanillic acid hexoside, catechin, naringenin, prunin, apigenin-*O*-hexoside, rutin, and rutin-*O*-pentoside was even much higher in byproducts than that in whole fruits. These results reveal the wide range of polyphenols found in tomato byproducts, with potential applications in pharmaceutical research, food preservation, and cosmetic development, among others.

## 1 Introduction

The tomato processing industry produces different residues/wastes with dangerous economic and environmental implications, such as release of greenhouse gases and billions of liters of water waste, loss of gross domestic product, etc.^1,2^ According to official data from the Food and Agriculture Organization of the United Nations (FAO, UN), tomato is the second most distributed horticultural crop worldwide, with a production rate of more than 186 million tons per year.^3^ However, it also generates around 8.5 million tons of byproducts, where peel and seeds represent 61% and 38%, respectively, of the total amount. Therefore, research on their exploitation is preconized to enforce the UN sustainable development agenda in the framework of circular economy.^4^

Despite that tomato byproducts have been mainly employed for animal feed or compost production, disposal of such wastes is currently a costly issue. However, applying tomato peel and seeds in the pharmaceutical, food, and cosmetic fields is a reality now. For example, tomato seed extract was recently commercialized as a nutritional supplement, claiming the improvement of sport performance in users;^5^ refined flour substituted with around 40% of tomato seeds exhibited higher amounts of dietary fiber and vitamin C, as well as longer shelf life, than those in traditional bakery products;^6^ addition of dried peel in raw and cooked beef burgers (at 4%) improved their sensorial and physicochemical characteristics like color and texture.^7^

Utilizing tomato byproducts in all mentioned areas is possible since peel and seeds are rich sources of bioactive compounds. Like tomato fruits, byproducts show exceptional concentrations of phytochemicals (carotenoids and polyphenols), vitamins (ascorbic acid, tocopherols, and provitamin A), glycoalkaloids (tomatine), pectin, fatty acids, and minerals.^8,9^ Coelho and coworkers have encouraged the integral valorization of tomato byproducts through the recovery of bioactive compounds, indicating that the mainly exploited compounds from tomato byproducts are carotenoids which account for around 70% of the total chemical composition of tomato matrices, but a promising minor fraction with exploitation potential includes phenolic compounds.^10^

The main body of studies on polyphenols from tomato byproducts has been focused on the extraction processing of such molecules and, a priori, their identification and quantification using liquid chromatography (LC) with UV detection or low-resolution mass spectrometry (LRMS). Ferreres and coworkers identified 14 flavonols in tomato seeds, including quercetin, kaempferol, and isorhamnetin derivatives, employing an LC-LRMS with an ion trap mass analyzer.^11^ On the other hand, Tamasi and coworkers reported six polyphenols in tomato peel using an LC-LRMS system with a triple-quadrupole mass analyzer, finding that caffeic acid, chlorogenic acid, and rutin were the most abundant polyphenols.^12^ By this way, Kalogeropoulos and coworkers carried out the most exhaustive identification panel of polyphenols, determining 18 polyphenols within phenolic acid and flavonoid families, where a selective ion monitoring (SIM) gas chromatography–mass spectrometry (GC-MS) method was used.^13^

The extensive knowledge of the extraction processing of polyphenols contrasts with the need for more information about the phenolic composition in tomato byproducts, which should be as broad as possible. López-Yerena reviewed and summarized the literature on identifying polyphenols in tomato byproducts and outlined their extraordinary exploitation potential in different industrial areas;^14^ however, the exhaustive polyphenolic profile has not yet been evaluated.

Thus, the main objective of this study was to characterize the polyphenolic profile in tomato peel and seeds by the analysis of hydroethanolic extracts using an untargeted analytical strategy based on LC coupled to high-resolution MS (LC-HRMS) with an LTQ Orbitrap analyzer. The combination of full-scan and data-dependent acquisition modes was employed to increase the detection coverage of polyphenols in the extracts. To understand the importance of their valorization, the polyphenolic profile obtained from these two byproducts was compared to the profile of the whole fruit, namely, a solid matrix with all components – like peel, pulp, seeds, and other tissues – which is regularly consumed fresh. For this purpose, an unsupervised multivariate analysis was carried out to find patterns that can be used to sum up differences and a univariate statistical analysis was then employed to profoundly investigate the changes on the leading phenolic families and representative polyphenols. This is the first report regarding the comprehensive elucidation of the phenolic composition of tomato peel and seeds, as well as the investigation of significant changes on the abundances of representative polyphenols in such byproducts with respect to the whole fruit. It was also intended to identify a higher number of phenolic compounds in these matrices in order to increase the knowledge of tomato composition. All these results had the goal to contribute with relevant information in the polyphenols present in these kinds of waste which could help to propose new routes for their valorization in the framework of circular economy.

## 2 Materials and Methods

### 2.1 Chemicals and Reagents

Standards of the following polyphenols were used for confirmation purposes: gallic acid, caffeic acid, ferulic acid, vanillic acid, syringic acid, 4-hydroxybenzoic acid, *p*-coumaric acid, protocatechuic acid, homovanillic acid, sinapic acid, apigenin, quercetin, and kaempferol were supplied by Sigma-Aldrich (St. Louis, MO, USA); catechin, rutin, and myricetin were provided by TCI (Tokyo, Japan); quercetin and chlorogenic acid were from Merck (Darmstadt, Germany); epigallocatechin and naringenin were from Biosynth Carbosynth (Berkshire, United Kingdom); diosmin was from Alfa Aesar (Kandel, Germany); galangin was from Cymit (Barcelona, Spain). A stock solution of each polyphenol was prepared in DMSO (Panreac, Barcelona, Spain) at a concentration of 5000 mg L^–1^. Working solutions for LC-HRMS analysis were prepared at 5 and 10 mg L^–1^ with acetonitrile/water (50/50, v/v) from the stock solutions.

For chromatographic separation, the following solvents were used: water purified with an Elix 3 coupled to a Milli-Q system (Bedford, USA), formic acid (≥95%, Sigma-Aldrich, St Louis, USA), and acetonitrile (99.9%, UHPLC Supergradient, Panreac, Barcelona, Spain).

### 2.2 Instrumentation and LC-HRMS Analysis

A Dionex UHPLC system coupled to an LTQ Orbitrap Velos mass spectrometer with an ESI-II electrospray ionization source (Thermo Scientific, Ca, USA) was used for the analysis of extracts.

The chromatographic separation was carried out with a Kinetex C18 column (150 mm length × 4.6 mm I·D, 2.6 μm partially porous particle size) from Phenomenex (Torrance, CA, USA) equipped with a SecurityGuard ULTRA cartridge C18 (Phenomenex). The mobile phase consisted of 0.1% (v/v) formic acid (solvent A) and acetonitrile (solvent B). A constant flow rate of 0.7 mL min^–1^ was used. The gradient elution program employed was as follows: initially, 3% B was maintained for 3 min, then, from 3% to 30% B was applied for the next 18 min, and the percentage of B was increased linearly to 65% from 18 to 23 min. Then, the percentage of B was increased up to 90% in 2 min and kept constant for additional 2.5 min. Finally, the percentage of B was decreased to initial conditions (3%) in 0.5 min, and the column was conditioned for 7 min before the next injection. The injection volume was 10 μL.

HRMS with the LTQ Orbitrap was carried out in negative full scan mode (from *m*/*z* 100 to 1500) using a resolution of 60 000 full-width at half-maximum (FWHM) at *m*/*z* 200. In addition, a data-dependent product ion scan was activated when the full scan signal was higher than 1.0 × 10^5^ (peak intensity threshold). Stepped normalized collision energies (NCEs) of 17.5, 35.0, and 52.5 were applied, and HRMS/MS spectra were recorded from an *m*/*z* of 50 Da. A mass resolution of 17 500 FWHM at *m*/*z* 200 was used for data-dependent analysis. Nitrogen (purity higher than 99.98%) was used as ESI sheath gas, ion-sweep gas, and auxiliary gas, at flow rates of 60, 0, and 10 arbitrary units, respectively. Capillary and S-lens RF voltages were set at −2.5 kV and 50 V, respectively. The source temperature was maintained at 25 °C, and the capillary temperature at 320 °C. The HRMS analyzer was tuned and calibrated every 3 days by using the calibration solution supplied by Thermo Fisher Scientific. Initially, the tentative identification (based on accurate mass errors below 5 ppm) of polyphenols was performed with fragmentation patterns from HRMS and HRMS/MS spectra; when available, they were compared with those of pure standards for a definitive confirmation.

LC-HRMS data were acquired and processed with Xcalibur 2.2 (Thermo Scientific, Ca, USA). The peak areas of the compounds from extracted ion chromatograms were integrated using OriginPro 8 software (OriginLab Corporation, USA). For the different statistical analyses, SOLO (eigenvector Research, USA), Statgraphics Centurion 19 (STATPOINT Inc., USA), and Microsoft Excel 2019 (Microsoft Corporation, USA) software were used.

### 2.3 Samples and Sample Treatment

Three sets of 2-kg oblong tomato fruit samples were acquired at local markets from Barcelona, Spain (September 2023); they were produced at two different Spanish regions (Murcia and Almería). The samples were treated within 24 h from purchase and cleaned with distilled water. After that, one set was pooled to represent an analytical sample of fruit, one for a peel sample, and one for a sample of seeds. This step was independently carried out for each region. All samples were frozen at −20 °C and lyophilized for 48 h using a freeze-dryer HT 40 from Telstar LyoQuest (Barcelona, Spain). Finally, samples were ground and stored at −20 °C in darkness until analysis.

Extraction of polyphenolic compounds from solid matrices was carried out using a solid–liquid procedure previously developed.^15^ Briefly, 0.5 g of sample were extracted with 30 mL of ethanol/water (75/25, v/v) at 40 °C under continuous stirring. Subsequently, extracts were centrifuged for 15 min at 3500 rpm and filtered in a 0.22-μm membrane, and then placed in 2 mL LC vials.

## 3 Results and Discussion

### 3.1 Examination of LC-HRMS Data

An outlook of total ion chromatograms from extracts is shown in Figure S1, with complex chemical fingerprints of all samples. The chromatographic elution profiles show two predominant elution windows; the first was from 4 to 15 min, attributed to polar features, and the second was in the range from 20 to 27 min, attributed to semipolar ones. Moreover, an elution window was detected between these ranges with few peaks. Thus, a total window, which involves the three mentioned, from 4 to 27 min, was chosen to extract the molecular features, whereby the most significant number of components could be evaluated, excluding death time.

The identification scheme of the molecular features was carried out as follows: possible phenolic constituents were first summarized based on published literature and available databases in terms of their chemical family, molecular formula, molecular mass, and fragment ion information. After that, total ion chromatograms were examined to detect peaks by matching with those of possible constituents, and extracted ion chromatograms were then used to obtain retention time, accurate mass, error, and HRMS/MS fragments of each feature.

As a result, the tentative identification of polyphenols was achieved by comparing the experimental HRMS data and the previously summarized information, and possible fragmentation pathways were elucidated employing characteristic fragments and neutral losses. Available polyphenol standards were analyzed to confirm the presence of some compounds, and the obtained HRMS/MS spectra were compared with those of the extracts.

Under this scheme, the total number of identified polyphenols was 57, and the assigned compounds and their LC-HRMS information are described in Table 1. To the best of our knowledge, from the whole list of polyphenolic compounds, it was the first time that epigallocatechin, arbutin, diosmin, diosmetin-*O*-hexoside, galangin 3-[galactosyl-(1→4)-rhamnoside], homovanillic acid, two coumaroyltartaric acid isomers, and dihydroferulic acid glucuronide were found in tomato or related matrices, whereby this paper contributes to increase the knowledge on the metabolic profile of those matrices.

**Table 1:** Polyphenolic Compounds Found in Hydroethanolic Extracts from Tomato Fruit, Peel, and Seeds and the Main LC-HRMS/MS Parameters that Support their Identification*

compound	chemical formula	retention time (min)	precursor ion *m*/*z* calculated value	precursor ion *m*/*z* observed value	adduct	error mass (ppm)	main MS/MS fragments
gallic acid^a^	C_7_H_6_O_5_	6.32	169.01314	169.01466	[M-H]^−^	2.446	125.02372
cinnamic acid	C_9_H_8_O_2_	6.78	193.04953	193.05109	[M-H+HCOOH]^−^	2.372	147.02982
							129.01892
							113.02410
							103.03966
hydroxybenzoic acid-*O*-hexoside	C_13_H_16_O_8_	8.01	299.07614	299.07790	[M-H]^−^	2.205	137.02432
							93.08644
vanillic acid^a^	C_8_H_8_O_4_	8.30	167.03388	167.03532	[M-H]^−^	2.023	152.01074
							123.04369
coumaroyltartaric acid isomer	C_13_H_12_O_8_	9.68	295.04592	295.04562	[M-H]^−^	–1.086	163.04472
							132.03430
							119.03445
							101.02397
vanillic acid hexoside	C_14_H_18_O_9_	9.74	329.08670	329.08650	[M-H]^−^	–3.966	285.09784
							167.04552
							123.04502
dihydroxybenzoic acid isomer I	C_7_H_6_O_4_	9.95	153.01823	153.01923	[M-H]^−^	–0.666	109.02962
arbutin	C_12_H_16_O_7_	10.56	317.08670	317.08847	[M-H+HCOOH]^−^	2.096	227.08145
							109.02852
monocaffeoylquinic acid isomer I (neochlorogenic acid)	C_16_H_18_O_9_	10.82	353.08670	353.08820	[M-H]^−^	1.118	191.05640
							179.03545
							173.04575
							135.04498
dihydroxybenzoic acid isomer II	C_7_H_6_O_4_	10.89	153.01823	153.01961	[M-H]^−^	1.817	109.02959
dihydroxybenzoic acid-*O*-pentoside	C_12_H_14_O_8_	11.10	285.06049	285.06107	[M-H]^−^	–1.826	153.01904
							109.02907
coumaric acid isomer I	C_9_H_8_O_3_	11.31	163.03897	163.04053	[M-H]^−^	2.837	119.04977
dihydroferulic acid glucuronide	C_16_H_19_O_10_	11.31	371.09727	371.0997	[M-H]^−^	3.584	325.09152
							163.03935
homovanillic acid-*O*-hexoside	C_15_H_20_O_9_	11.65	343.10235	343.10452	[M-H]^−^	3.103	181.05069
							137.06046
eriodictyol	C_15_H_12_O_6_	11.73	287.05501	287.05530	[M-H]^−^	–2.826	151.00370
							135.04545
							125.02450
4-hydroxybenzoic acid^a^	C_7_H_6_O_3_	11.78	137.02332	137.02403	[M-H]^−^	–2.827	93.03410
caffeic acid-*O*-hexoside	C_15_H_17_O_9_	11.81	341.0867	341.08746	[M-H]^−^	–1.012	179.03436
							135.04460
catechin^a^	C_15_H_14_O_6_	12.15	289.07066	289.07839	[M-H]^−^	1.036	245.07366
							203.05232
							123.04862
apigenin-*O*-hexoside isomer I	C_21_H_20_O_10_	12.25	431.09727	431.09726	[M-H]^−^	–2.575	269.04119
							225.05496
							175.01565
coumaric acid-*O*-hexoside	C_15_H_17_O_8_	12.45	325.09179	325.09430	[M-H]^−^	4.335	163.03976
							119.04962
protocatechuic acid^a^	C_7_H_6_O_4_	12.52	153.01823	153.01878	[M-H]^−^	–0.552	109.02956
homovanillic acid^a^	C_9_H_10_O_4_	12.82	227.05501	227.05516	[M-H]^−^	–4.190	137.05551
monocaffeoylquinic acid isomer II (cryptochlorogenic acid)	C_16_H_18_O_9_	13.11	353.08670	353.08734	[M-H]^−^	–1.318	191.05614
							179.03503
							173.04565
							135.04512
apigenin-*O*-hexoside isomer II	C_21_H_20_O_10_	13.56	431.09727	431.09726	[M-H]^−^	–2.575	269.04095
							225.05487
							175.01572
ferulic acid-*O*-hexoside	C_16_H_20_O_9_	13.68	355.10235	355.10269	[M-H]^−^	–2.155	193.05016
							178.04008
							149.06064
caffeic acid^a^	C_9_H_8_O_4_	13.69	179.03388	179.03522	[M-H]^−^	1.329	135.04501
epigallocatechin^a^	C_15_H_14_O_7_	13.76	305.06557	305.06645	[M-H]^−^	–1.593	305.06123
							125.02442
							109.06123
monocaffeoylquinic acid isomer III (chlorogenic acid a)	C_16_H_18_O_9_	14.00	353.08670	353.08759	[M-H]^−^	–0.610	191.05566
							179.03439
4-hydroxybenzoic acid isomer	C_7_H_6_O_3_	14.13	137.02332	137.02414	[M-H]^−^	–2.024	93.03695
syringic acid^a^	C_9_H_10_O_5_	14.13	197.04444	197.04588	[M-H]^−^	1.692	182.05101
							153.05028
coumaroyltartaric acid isomer II	C_13_H_12_O_8_	15.11	295.04592	295.04562	[M-H]^−^	–1.086	163.04489
							132.03421
							119.03456
							101.02384
myricetin^a^	C_15_H_10_O_8_	15.36	317.02919	317.02917	[M-H]^−^	–3.534	301.04257
							273.03082
							151.00361
coumaroylquinic acid	C_16_H_18_O_8_	15.75	337.09179	337.09225	[M-H]^−^	–1.901	191.05507
							163.04123
rutin-*O*-pentoside	C_32_H_38_O_20_	15.87	741.18726	741.18781	[M-H]^−^	NA	609.14453
							301.03421
							300.02655
							178.99792
kaempferol-*O*-hexoside	C_21_H_20_O_11_	16.49	447.09218	447.09283	[M-H]^−^	–1.017	285.03851
							175.03975
galangin 3-[galactosyl-(1→4)-rhamnoside]	C_27_H_30_O_14_	16.52	577.15518	577.15698	[M-H]^−^	1.215	341.10760
							269.04443
							179.05547
							161.04498
*p*-coumaric acid^a^	C_9_H_8_O_3_	16.76	163.03897	163.04019	[M-H]^−^	0.752	119.05045
rutin^a^	C_27_H_29_O_16_	16.86	609.14501	609.14514	[M-H]^−^	NA	301.03452
							300.02689
							178.99789
phloretin-*C*-diglucoside	C_27_H_34_O_15_	16.93	597.18139	597.18219	[M-H]^−^	–0.508	597.18159
							477.18023
							273.07483
sinapic acid^a^	C_11_H_12_O_5_	17.53	223.06009	223.06059	[M-H]^−^	–2.720	208.03522
							179.05569
quercetin-*O*-hexoside isomer I	C_21_H_19_O_12_	17.53	463.08710	463.08917	[M-H]^−^	2.096	301.03343
							300.99658
							178.99796
							151.00313
kaempferol-*O*-rutinoside	C_27_H_30_O_15_	17.99	593.15009	593.15002	[M-H]^−^	–1.978	431.11533
							285.03867
							175.03958
naringenin-*O*-hexoside (Prunin)	C_21_H_22_O_10_	18.82	433.11292	433.11304	[M-H]^−^	–2.263	271.08127
							177.08153
							161.04480
diosmin^a^	C_28_H_32_O_15_	18.92	607.16574	607.16608	[M-H]^−^	–1.257	299.05726
							151.00312
diosmetin-*O*-hexoside	C_22_H_22_O_11_	19.28	461.10783	461.10809	[M-H]^−^	–1.832	299.05618
							151.00365
							149.02641
dicaffeoylquinic acid isomer I	C_25_H_24_O_12_	19.36	515.11840	515.12158	[M-H]^−^	4.039	353.08768
							191.05626
							173.04650
kaempferol isomer	C_15_H_10_O_6_	21.45	285.03936	285.03995	[M-H]^−^	–1.794	285.03969
							175.03948
							151.00324
quercetin-*O*-hexoside isomer II	C_21_H_20_O_12_	21.78	463.08710	463.08737	[M-H]^−^	–1.791	301.03345
							300.99658
							178.99796
							151.00325
dicaffeoylquinic acid isomer II	C_25_H_24_O_12_	21.85	515.11840	515.12158	[M-H]^−^	4.039	353.08779
							191.05615
							173.04642
quercetin^a^	C_15_H_10_O_7_	22.12	301.03427	301.03598	[M-H]^−^	2.007	300.99658
							178.99796
							151.00313
							149.02365
kaempferol^a^	C_15_H_10_O_6_	22.31	285.03936	285.03995	[M-H]^−^	–1.794	285.03955
							175.03938
							151.00310
naringenin^a^	C_15_H_12_O_5_	23.12	271.06009	271.06122	[M-H]^−^	0.086	271.06095
							177.08135
							161.04358
							151.03003
							119.00356
apigenin^a^	C_15_H_10_O_5_	23.21	269.04444	269.04522	[M-H]^−^	–1.214	269.04801
							225.05506
							175.01542
							151.00226
							149.02408
quercetin isomer	C_15_H_10_O_7_	23.25	301.03427	301.03482	[M-H]^−^	–1.847	300.99672
							178.99782
							151.00329
							149.02381
apigenin isomer	C_15_H_10_O_5_	23.80	269.04444	269.04514	[M-H]^−^	–1.512	269.04801
							225.05506
							151.00226
							149.02408
ferulic acid^a^	C_10_H_10_O_4_	24.23	193.04953	193.05039	[M-H]^−^	–1.254	178.04015
							149.04507
isorhamnetin	C_16_H_12_O_7_	24.57	315.04992	315.04953	[M-H]^−^	2.679	315.04912
							151.00325
							119.00263

a Polyphenolic compounds confirmed by LC-HRMS analysis of reference standards.

* NA: not applicable.

Furthermore, three peaks with high abundance were found but could not be assigned to any known compound. The peak at 6.92 min showed a precursor ion at *m*/*z* 164.07195 and fragments at *m*/*z* 147.04472 and 120.05328 in its HRMS/MS spectra; given masses correspond to Δ*m*/*z* 17 and 44, attributed to dihydroxylation and decarboxylation, respectively, which are typical for phenolic acids. The peak at 18.19 min showed a precursor ion at *m*/*z* 741.19135 and HRMS/MS fragments at *m/z,* 807.31563, 779.61233, 747.36616, and 682.33643. Finally, the peak at 26.10 showed a precursor ion at *m*/*z* 353.20111 with HRMS/MS fragments at *m*/*z* 352.20041, 332.20013, 302.23311, and 122.20031. This information is for further studies that could be carried out to elucidate the molecular structure of the found compounds using a battery of spectroscopic techniques.

In the following sections, the characteristic fragmentation patterns supporting the identification of the detected phenolic compounds are discussed with various meaningful cases (see the Supporting Information).

### 3.2 Identification of Phenolic Acids and Derivatives

12 hydroxybenzoic acids and 18 hydroxycinnamic acids were detected, most of them first assigned to their [M-H]^−^ ions in HMRS full scan and by the monitoring of the decarboxylation process in the HRMS/MS spectra as primary fragmentation.^16,17^ For instance, the peak at 6.32 min showed a *m*/*z* value of 169.01466, which matched the gallic acid [M-H]^−^ ion with an error of 2.446 ppm. In the analysis of HRMS/MS spectra (Figure S2A), a single ion of *m*/*z* 125.02372 was detected, which corresponds to the loss of the carboxylic moiety as −COO (Δ*m*/*z* = 44). The retention time and HRMS/MS spectra (Figure S2B) agree with those obtained in the pure standard, confirming the identification of this compound as gallic acid. In the same way, two hydroxybenzoic acid isomers, three dihydroxybenzoic acid isomers, two coumaric acid isomers, syringic acid, ferulic acid, and caffeic acid were assigned.

In another illustrative case, the HRMS full-scan spectra of the chromatographic peak at 12.99 min showed an ion of *m*/*z* 223.06059 with an error of −2.720 ppm, tentatively matching with the sinapic acid [M-H]^−^ chemical formula. As shown in Figure S3A, an ion of *m*/*z* 179.05569 was found in its HRMS/MS spectrum, corresponding to the decarboxylation process, and an ion of *m*/*z* 208.03522 was also detected which corresponds to the demethylation −CH_3_ (Δ*m*/*z* = 15) of the structure.^18^ When the pure standard was analyzed, retention time and MS/MS fragments were the same as those detected in the extracts (Figure S3B); thereby, the assignment of sinapic acid was confirmed. Analogously, vanillic acid was confirmed in the extracts.

**Figure 1:**
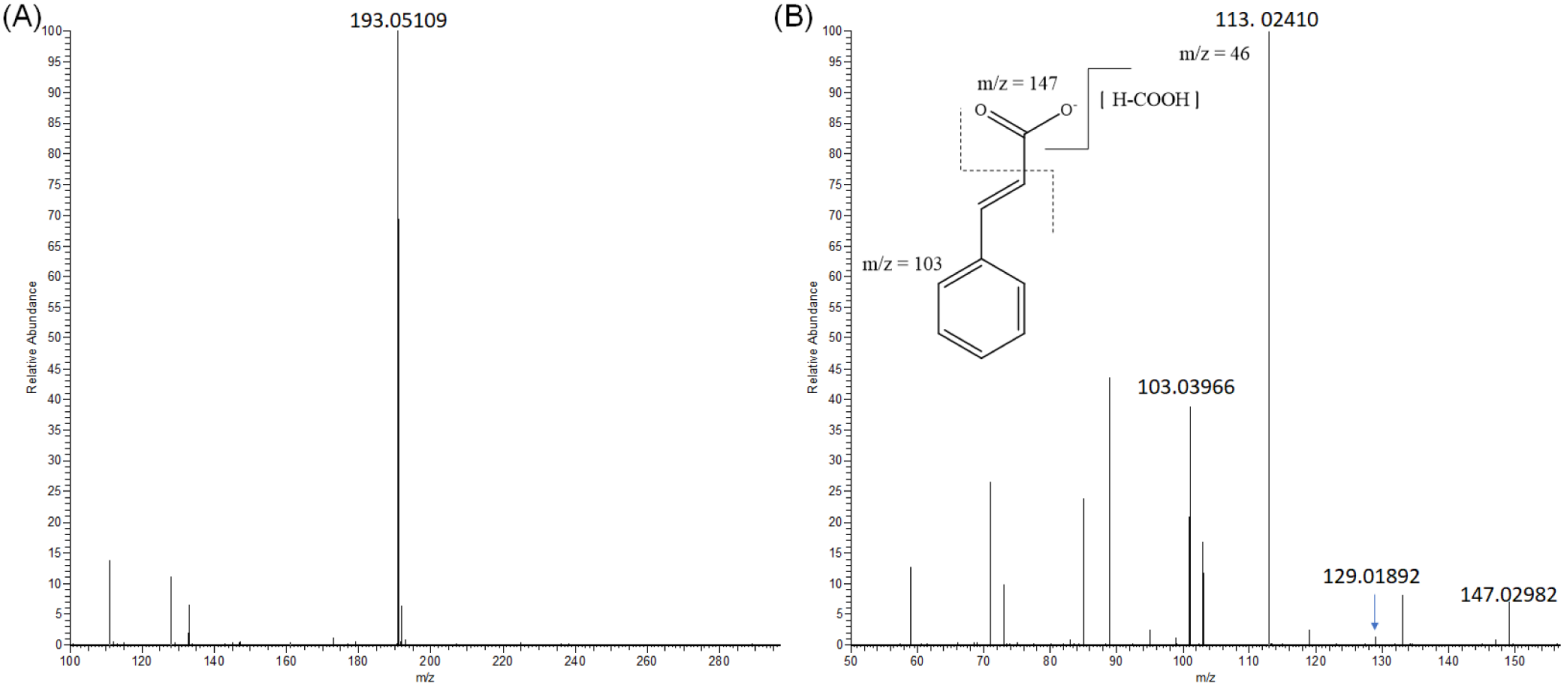
HRMS spectra (A) and HRMS/MS spectra (B) of cinnamic acid from tomato and byproducts extracts. Dotted line indicates a fragment of ion at *m*/*z* 147.

Although [M-H]^−^ is the main ion of phenolic compounds in negative ESI, the [M-H+HCOOH]^−^ adduct could be potentially detected.^19^ For example, the peak at 6.78 min with the HRMS spectra (Figure 1A) showed a precursor ion of *m*/*z* 193.05109 and with an error of 2.372 ppm, which was not primarily assigned to any compound; however, in the HRMS/MS spectra (Figure 1B), an ion at *m*/*z* 147.02982 was detected and matched with a molecular formula of the cinnamic acid moiety. Besides, two ions at *m*/*z* 129.01892 and *m*/*z* 103.03966 were observed, which are neutral losses of −H_2_O (Δ*m*/*z* = 18) and −COO, respectively, so this species was tentatively identified as cinnamic acid. For arbutin, this fragmentation pattern was also observed; the peak at 10.56 min showed HRMS spectra (Figure S4A) with a precursor ion at *m*/*z* 317.08847 which matches with [M-H+HCOOH]^−^ of arbutin (error: 2.096 ppm). During the exploration of its HRMS/MS spectra (Figure S4B), it was noted that a fragment ion at *m*/*z* 227.08145 corresponded to formula C_12_H_15_O_7_ for arbutin, and an ion at *m*/*z* 109.02852 corresponded to the catechol moiety after the neutral loss of hexoside. This fragmentation pattern was like that proposed by Song and coworkers.^20^

Another fragmentation that could be found is that in the de-esterification; this is the cleavage of *O*-linkage between a phenolic acid moiety and a glycoside moiety, or two phenolic acid moieties, with different similar structures.^21−23^ In this sense, eight glycoside derivatives, three monocaffeoylquinic acid isomers, two dicaffeoylquinic acid isomers, two coumaroyltartaric acid isomers, and a monocoumaroylquinic acid were identified in the extracts.

For the assignation of glycoside derivatives, neutral losses of hexose (Δ*m*/*z* = 162), pentose (Δ*m*/*z* = 132), rhamnose (Δ*m*/*z* = 146), and glucuronide (Δ*m*/*z* = 176) moieties were investigated as the main fragmentations.^17^ An example of this is the peak at 11.10 min; this showed an ion at *m*/*z* 285.06107 in HRMS spectra, resulting in a chemical formula of C_12_H_13_O_8_ with an error of −1.826 ppm. Exploration of its HRMS/MS spectra (Figure S5A) led to relayed in ions at *m*/*z* 153.01904 and *m*/*z* 109.02907, meaning neutral losses of −C_5_H_8_O_4_ (Δ*m*/*z* = 132) and −COO, respectively, and matching with the fragmentation pattern of dihydroxybenzoic acid-*O*-pentoside. Similarly, homovanillic acid-*O*-hexoside was elucidated. The HRMS spectra of the peak at 11.65 min denoted an ion at *m*/*z* at 343.10452 with C_15_H_19_O_9_ as the proposed formula and an error of 3.103 ppm, and the HRMS/MS spectra of this ion (Figure S5B) showed fragments of *m*/*z* 181.05069 and *m*/*z* 137.06046, representing −C_6_H_10_O_5_ (Δ*m*/*z* = 162) and −COO, respectively, and matching with the fragmentation pattern of that polyphenol.

Coumaroyltartaric acid (coutaric acid) isomers were identified as follows: peaks at 9.68 and 15.11 min, which showed [M-H]^−^ of *m*/*z* 295.04562 with an error of −1.086 ppm (Figure S6A), corresponded to the loss of the tartaric acid moiety (Δ*m*/*z* = 132) and the base peak of the coumaric acid moiety (*m*/*z* 163.04472) in their HRMS/MS spectra (Figure S6B).^24^ Besides, the ion at *m*/*z* 119.03445 was observed in that spectra because of the neutral loss of −COO in the coumaric acid moiety, and then, the ion at *m*/*z* 101.02397 can be attributed to structural rearrangement of such an ion by −H_2_O loss.

In the case of monocaffeoylquinic acid isomers, peaks at 10.82, 13.11, and 14.00 min had [M-H]^−^ of *m*/*z* 353.08820, 353.08734, and 353.08759, with errors of 1.118, −1.318, and −0.610 ppm, respectively, proposing C_16_H_17_O_9_ as the formula. It could be observed ions at *m*/*z* 191, 179, 173, and 135, which correspond to the quinic acid moiety, caffeic acid moiety, −H_2_O loss of the quinic acid moiety, and −COO loss of the caffeic acid moiety, respectively. According to the literature,^25,26^ three main isomers, chlorogenic, cryptochlorogenic, and neochlorogenic acids, can be distinguished by comparing the relative intensity of those ions. For the compound at 10.82 min (Figure S7A), the ratio of *m*/*z* 191 and 179 was approximately 100/20, while the ratio of *m*/*z* 191 and 135 was 100/30, conjecturing the presence of neochlorogenic acid. For the compound at 13.11 min (Figure S7B), the ion at *m*/*z* 173 showed 100-% intensity, being very specific for cryptochlorogenic acid. Finally, in the HRMS/MS spectra of compound at 14.00 min (Figure S7C), it was noticed that a ratio of *m*/*z* 191 and 179 was 100/<10, and this information matched with retention time and HRMS/MS spectra ions when the chlorogenic acid standard was analyzed (Figure S7D).

### 3.3 Identification of Flavonoids and Derivatives

Additionally, flavonoids were identified utilizing their characteristic fragments, attributed to the rupture of the B ring bond and the retro Diels–Alder fragmentation (*m*/*z* 151).^17,27^ Neutral losses like de-esterification can also be found by rupture of glycoside bonds and ruptures of −CO (Δ*m*/*z* = 28) and −CH_3_ (Δ*m*/*z* = 15).^28,29^

Peaks at 17.53 min showed a precursor ion at *m*/*z* 463.08917 with the formula of C_21_H_18_O_12_ (error: 2.096 ppm), whereas the precursor ion of the peak at 22.12 min was at *m*/*z* 301.03598 with the formula of C_21_H_18_O_12_ (error: 2.096 ppm). As shown in Figure S8, HRMS/MS spectra of ions at *m*/*z* 463 and *m*/*z* 301 show fragments at *m*/*z* 300.99658, 178.99796, and 151.00313. For the compound at 17.53 min, a difference of *m*/*z* 162 (−C_6_H_10_O_5_) was found, determining a rupture of the hexoside bond. In both compounds, spectra showed fragments at *m*/*z* 300.99658, 178.99796, 151.00313, and 149.02365; all these results matched with retention time and fragments found in the HRMS/MS spectra of quercetin standard solution (Figure S8C), so this compound was assigned as quercetin, showing a quercetin isomer and two quercetin-*O*-hexosides.

In addition, apigenin was found in peak at 23.21 min, which showed the precursor ion at *m*/*z* 269.04522 with the formula of C_15_H_9_O_5_ (errors: −1.214 and 1.512 ppm). HRMS/MS spectra are shown in Figure S9A. Fragments at *m*/*z* 269.04801, 225.05506, 175.01542, 151.002265, and 149.02408 were correlated with the fragmentation patterns of apigenin observed by Chiriac^29^ and Kečkeš.^30^ The fragment at *m*/*z* 175 was related explicitly to the rupture of bond of phenol-type B ring in apigenin structure. Furthermore, retention times and fragments matched with HRMS/MS spectra from apigenin standard (Figure S9B).

**Figure 2:**
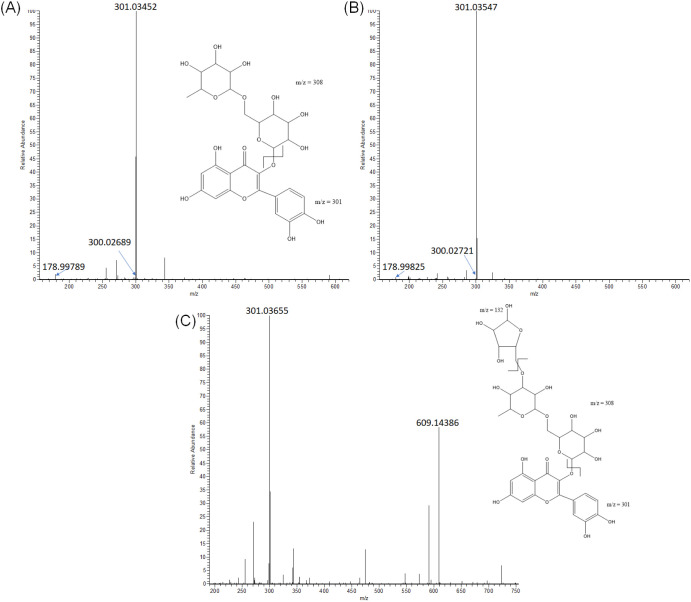
HRMS/MS spectra of rutin from tomato and byproducts extracts (A), rutin standard solution (B), and rutin-*O*-pentoside (C) from tomato and byproducts extracts.

On the other hand, a particular case was the identification of rutin and rutin-*O*-pentoside. HRMS spectra of the peak at 16.86 min showed a precursor ion at *m*/*z* 609.14514 which did not match with any chemical formula; however, during the visualization of its HRMS/MS spectrum (Figure 2A), we observed ions at 301.03452 and 300.02689 which mean [M-H]^−^ and [M-2H]^−^ of quercetin with Δ*m*/*z* = 308 due to the rupture of rutinoside bond, as well as fragment at *m*/*z* 178. The retention time and HRMS/MS fragmentation pattern (Figure 2B) were in concordance with those of the rutin standard. Likewise, peak at 15.87 min with a precursor ion at *m*/*z* 741.18781 did not match with any chemical formula, but HRMS/MS shown in Figure 2C enabled the detection of ions at *m*/*z* 609.14453, with Δ*m*/*z* = 132 attributed to the rupture of the pentoside bond, and at *m*/*z* 301.03421 and 300.02655, which are parallel to data obtained in the HRMS/MS spectra of rutin, and enabled the assignation of this compound such as rutin-*O*-pentoside.

### 3.4 Unsupervised Exploratory Analysis

To evaluate differences between the polyphenolic profiles in the six classes of samples under study, an unsupervised principal component analysis (PCA) was conducted using a data set constructed from peak abundances (peak areas in extracted ion chromatograms) of the 57 found polyphenolic compounds. Data were preprocessed by autoscaling, and the number of principal components (PC) was set at 3.

**Figure 3:**
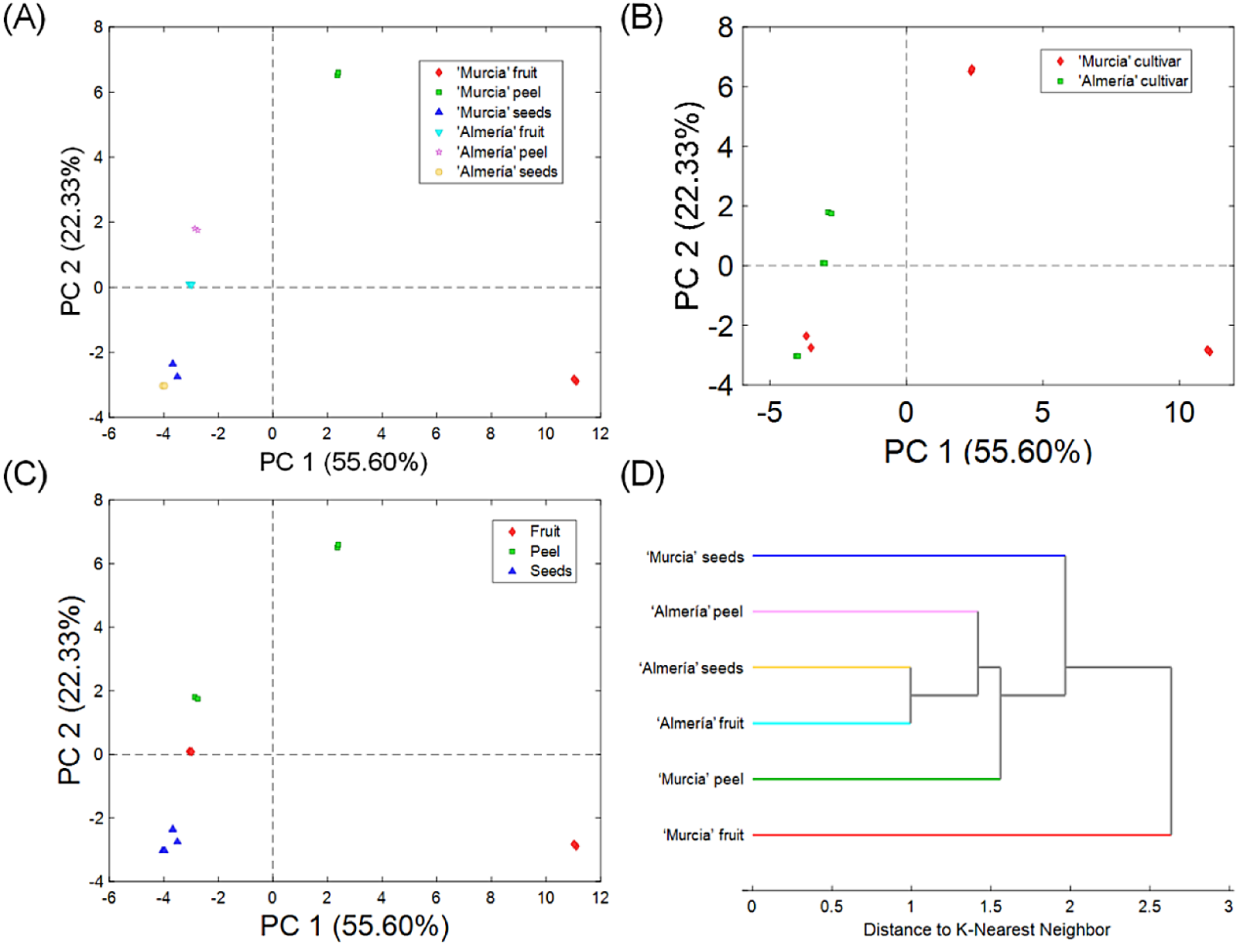
Unsupervised exploration of polyphenolic profiles in samples by use of principal component analysis and hierarchical clustering analysis. Score plots from the principal component analysis of samples divided in 6 different classes (A), regions (B), and groups (C). Dendrogram plot from hierarchical clustering analysis for the 6 classes of samples (D). Data set was formed with peak abundances of the 57 identified polyphenolic compounds in LC-HRMS/MS.

The obtained model explains a total accumulative variance with a *Q*-residuals value of 10.99% and a Hotelling *T*^2^ value of 89.01%. PC1 and PC2 support 55.60% and 22.33% of variance, respectively. When data were analyzed along the six different classes of samples (Figure 3A), the complete discrimination of’Murcia’ fruit and peel was done in PC1, whereas’Murcia’ peel,’Almería’ peel, and’Almería’ fruit were differentiated, along PC2. However, the PCA did not discriminate all the samples between the two analyzed regions (Figure 3B). On the other hand, a study of the differences between fruit, peel, and seeds, without the dependency of regions, showed a clear tendency of agglomeration for seeds (Figure 3C). The loadings revealed 14 polyphenolic compounds responsible for the discrimination among samples, including 4-hydroxybenzoic acid, a dihydroxybenzoic acid isomer, homovanillic acid-*O*-hexoside, caffeic acid, 4-O-caffeoylquinic acid, *p*-coumaric acid, dihydroferulic acid-*O*-glucuronide, eriodictyol, prunin, diosmetin-*O*-hexoside, quercetin, rutin, kaempferol, and phloretin-*C*-dihexoside.

The hierarchical clustering analysis (HCA) with *k*-nearest neighbors using results from the PCA set at the Mahalanobis distance enables an understanding of the found differences. The resulted dendrogram is shown in Figure 3D. The analysis separated the samples into three main groups. The “Murcia” fruit was the most different sample, where the abundances of the detected phenolic compounds were extensively different to the rest of the samples. Also, the total number of elucidated polyphenolic compounds was found in this sample. Besides, it was noticed that the abundances on all’Almería’ samples were different to “Murcia” samples, enabling a differentiation of both classes. The exploratory results indicate significant changes on their polyphenolic profiles, mainly attributed to the class of sample (fruit, peel, or seed).

### 3.5 Changes in the Abundances of the Main Phenolic Families between Tomato Fruit, Peel, and Seeds from “Murcia” and “Almería” Regions

Apart from the information obtained in exploratory analysis, the variations in the abundances of phenolic families were studied to deeply evaluate the differences in the polyphenolic profiles in the whole set of samples. The 57 polyphenols were classified into eight phenolic families (Table S1), and the total abundance of a phenolic family was estimated by the sum of the peak areas of all compounds belonging to such family. With the given scope in mind, data were subjected to one-way ANOVA at 95-% confidence, and then the Tukey HSD test was performed.^31^

**Figure 4:**
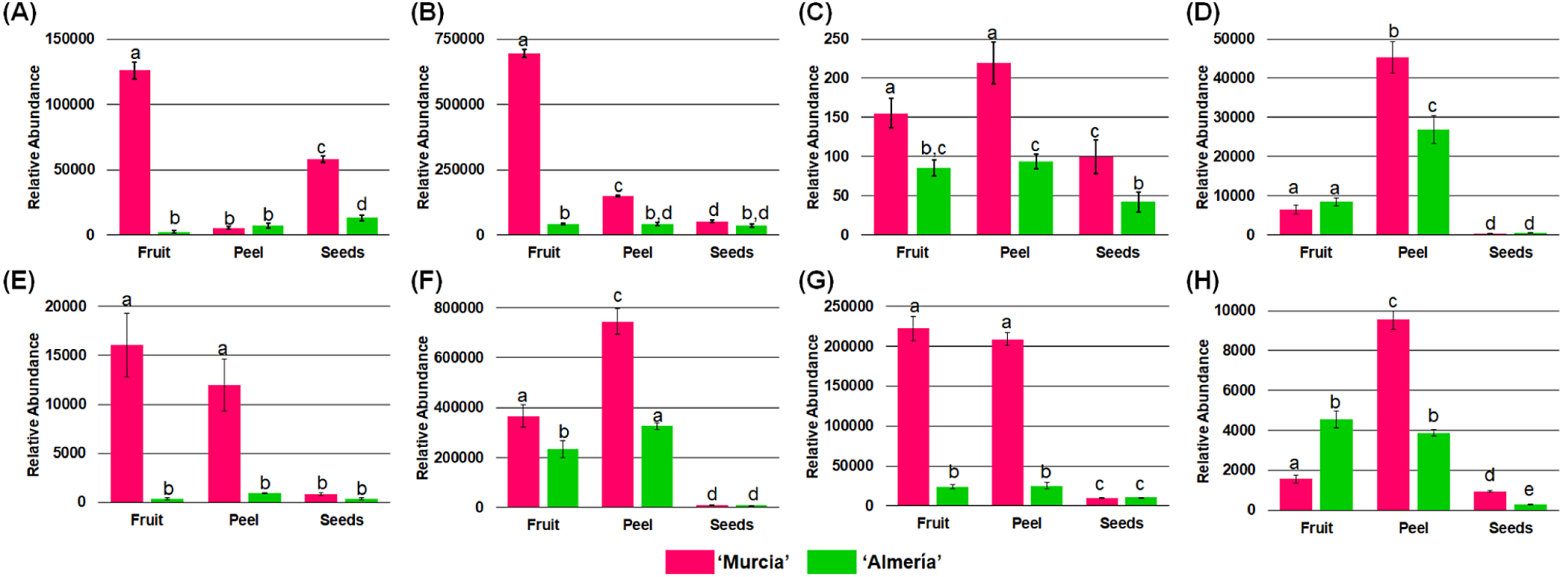
Relative abundances of the main phenolic families in tomato fruit, peel, and seeds from “Murcia” and “Almería” regions. (A) Hydroxybenzoic acids, (B) hydroxycinnamic acids, (C) flavanols, (D) flavanones, (E) flavones, (F) flavonols, (G) chalcones, and (H) other phenols.

Figure 4 shows the results of the distribution of the families among the samples. As the graphs imply, both phenolic acids were 4-fold higher in “Murcia” fruit than those in its respective peels and 2-fold higher than those in seeds. Contrarily, “Almería” samples showed different tendencies; peel showed a 50% increase in the content of hydroxycinnamic acids compared to seeds and fruit, but hydroxybenzoic acids were 1.6 times more concentrated in seeds than those in the other samples. All flavonoid classes show better distribution for both “Murcia” and “Almería” peels (up to 5 times concentrated) than those in respective fruit and seeds; only slight discrepancies, as in the case of flavones, were observed where this family was 50% more concentrated in “Murcia” fruit than that in the respective peel. In a similar way, other phenols were up to 3 times more distributed in peel than those in fruit and seeds for both regions.

The high abundance of these phenolic families in peel is well justified due to its role in attracting pollinator insects and protecting against biotic and abiotic stresses.^32^ Other phenols did not contribute significantly to the phenolic composition of any sample, and this family was more abundant in “Murcia” peel. The results show that tomato byproducts are excellent sources of phenolic acids and flavonols.

### 3.6 Changes in the Abundances of the Representative Polyphenols between Tomato Fruit, Peel, and Seeds from “Murcia” and “Almería” Regions

**Figure 5:**
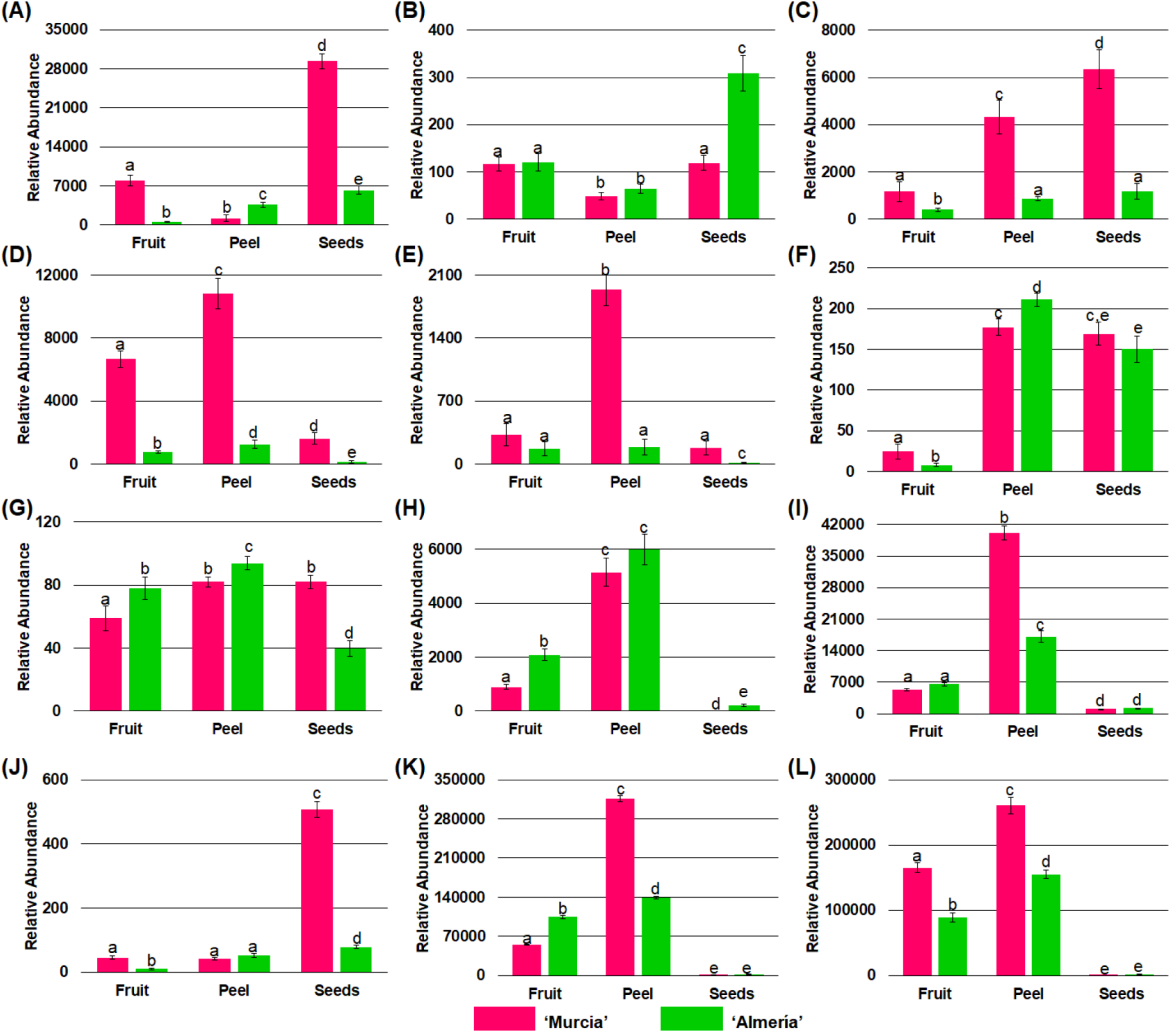
Relative abundances of polyphenolic compounds in tomato fruit, peel, and seeds from “Murcia” and “Almería” regions. (A) Vanillic acid hexoside, (B) coutaric acid isomer, (C) caffeic acid, (D) neochlorogenic acid, (E) dicaffeoylquinic acid, (F) ferulic acid, (G) catechin, (H) naringenin, (I) prunin, (J) apigenin-*O*-hexoside, (K) rutin, and (L) rutin-*O*-pentoside.

After evaluating the behavior of the phenolic families, the abundances of some representative polyphenols were also tested through comparing their peak abundance area obtained in extracted ion chromatograms by statistical analysis as indicated in the previous case. While’Murcia’ fruit has the highest concentration of most of the identified polyphenols, the principal goal of this work is to demonstrate the capability of tomato byproducts like enriched sources of bioactive polyphenols; thus, the studied polyphenols in this section were selected because they showed the highest content in any byproduct sample, and the results are depicted in Figure 5.

Vanillic acid hexoside (Figure 5A) was the hydroxybenzoic acid with more content in “Murcia” seeds, where the difference between the abundances of those samples was 97.27%; this compound was also more concentrated in “Almería” seeds with an increase of 98.48% and 42.22% in comparison to that in fruit and peel samples, respectively. The presence of this compound in tomato byproducts is reported for the first time herein. However, vanillic acid was previously detected in tomato wastes from industries after processing, where it can be hypothesized that employed processes could promote the rupture of the hexoside bonds, producing the release of aglycone form.^13,33,34^

Regarding hydroxycinnamic acids, five compounds were more abundant in tomato byproducts than those in the respective whole fruit: coutaric acid isomer (Figure 5B), caffeic acid (Figure 5C), neochlorogenic acid (Figure 5D), dicaffeoylquinic acid (Figure 5E), and ferulic acid (Figure 5F). Coutaric acid was 61.17% more concentrated in’Almería’ seeds than that in’Murcia’ and’Almería’ fruits and’Murcia’ peel. Caffeic acid showed a tendency where the abundance increased in the order of fruit < peel < seeds for both regions, with’Murcia’ byproducts overcoming up to 81.43% of the abundance of’Almería’ byproducts. In addition, this compound is the most detected one in a wide range of tomato byproduct samples from different environmental origins.^12,13,34−38^ By this way, the abundance of neochlorogenic acid in’Murcia’ peel was 1.57 times higher than that in the respective fruit. These phenolic acid and other monocaffeoylquinic acid isomers have been identified in tomato byproducts according to the literature.^12,13,33,35−37,39−41^ “Murcia” peel was notably the sample with the highest content of dicaffeoylquinic acid, with 83.18% more abundance than that in other samples. It is relevant to mention that coutaric acid and dicaffeoylquinic acid were found in tomato byproducts for the first time. In the case of ferulic acid, the concentration of this polyphenol in peel and seeds from the two regions was highly superior in comparison to the respective fruits (∼98.57%), and, specifically, peel was around 30% more concentrated than seeds. Ferulic acid is a polyphenol found in tomato byproducts from markets, cultivars, and factories.^13,34,36,37,42^

For flavonoids, catechin (Figure 5G) was slightly (12.76%) more abundant in “Almería” peel than other samples, and it has been described that its abundance is low in comparison with other polyphenols.^13,34,36,38^ On the other hand, a high abundance of naringenin and quercetin, as well as their derivatives like prunin and rutin, in tomato seeds and peel was reported before.^11,43,44^ Naringenin (Figure 5H) and prunin (Figure 5I) showed a higher abundance in peel from both regions than those in the whole fruit, with increases of 65.08% and 83.61%, respectively. For the first time, apigenin-*O*-hexoside (Figure 5J) was found in tomato byproducts, exceptionally concentrated in’Murcia’ seeds (∼90%), compared to that in other samples. Concerning rutin (Figure 5K) and rutin-*O*-pentoside (Figure 5L), their maximum abundance was observed in “Murcia” peel, even though it was up to 45% higher than that in “Almería” peel. Rutin has also been extensively found in tomato byproducts,^2,12,33,34,36−38,40,45,46^ and a rutin derivative was reported before, but its identity is not clear.^37^

To sum up, tomato peel and seeds – from two Spanish cultivars – contained a wide variety of polyphenolic compounds, where a significant number of them was found in these kinds of samples for the first time. Of the 57 detected compounds, most of them belong to phenolic acids and flavonoids, mainly flavonols. Among the most abundant compounds found in the byproducts, seven aglycones (coutaric acid, caffeic acid, neochlorogenic acid, dicaffeoylquinic acid, ferulic acid, catechin, and naringenin) and five glycoside derivatives (vanillic acid hexoside, prunin, apigenin-*O*-hexoside, rutin and rutin-*O*-pentoside) stand out. In this context, this paper offers relevant information to a wide range of people, like tomato producers, environmentalists, and other scientists, interested in the comprehensive valorization of tomato wastes through obtaining high-value natural products with elevated bioactive properties from these matrices.

## Supporting Information Available

Total ion chromatograms of all extracts with LC-HRMS, HRMS, and HRMS/MS spectra for representative polyphenolic compounds; classification of identified polyphenolic compounds (PDF)
