# Supplementary material for: Characterization of the Polyphenolic Profile in Tomato (Lycopersicon esculentum P. Mill) Peel and Seeds by LC-HRMS/MS
Source: J Agric Food Chem. 2024 Jul 8;72(28):15680–92. doi: 10.1021/acs.jafc.4c02126 (PMC11261606; doi:10.1021/acs.jafc.4c02126)
Supplement: Supplementary file 1 — jf4c02126_si_001.pdf [file jf4c02126_si_001.pdf]

SUPPORTING INFORMATION

Characterization of the polyphenolic profile in tomato (*Lycopersicon esculentum* P. Mill) peel and seeds by LC-HRMS/MS

Jared Mauricio López-Téllez[1,2,*], María del Pilar Cañizares-Macías[1], Aina Mir[2,3], Javier Saurina[2,3], Oscar Núñez[2,3,4,*]

[1]Department of Analytical Chemistry, Faculty of Chemistry, Universidad Nacional Autónoma de México, Mexico City, 04510, Mexico

[2]Department of Chemical Engineering and Analytical Chemistry, Universitat de Barcelona, Martí i Franquès 1-11, E08028 Barcelona, Spain

[3]Research Institute in Food Nutrition and Food Safety, Universitat de Barcelona, Av. Prat de la Riba 171, Edifici Recerca (Gaudí), E08921 Santa Coloma de Gramenet, Spain

[4]Serra Húnter Fellow Programme, Generalitat de Catalunya, Via Laietana 2, E08003 Barcelona, Spain

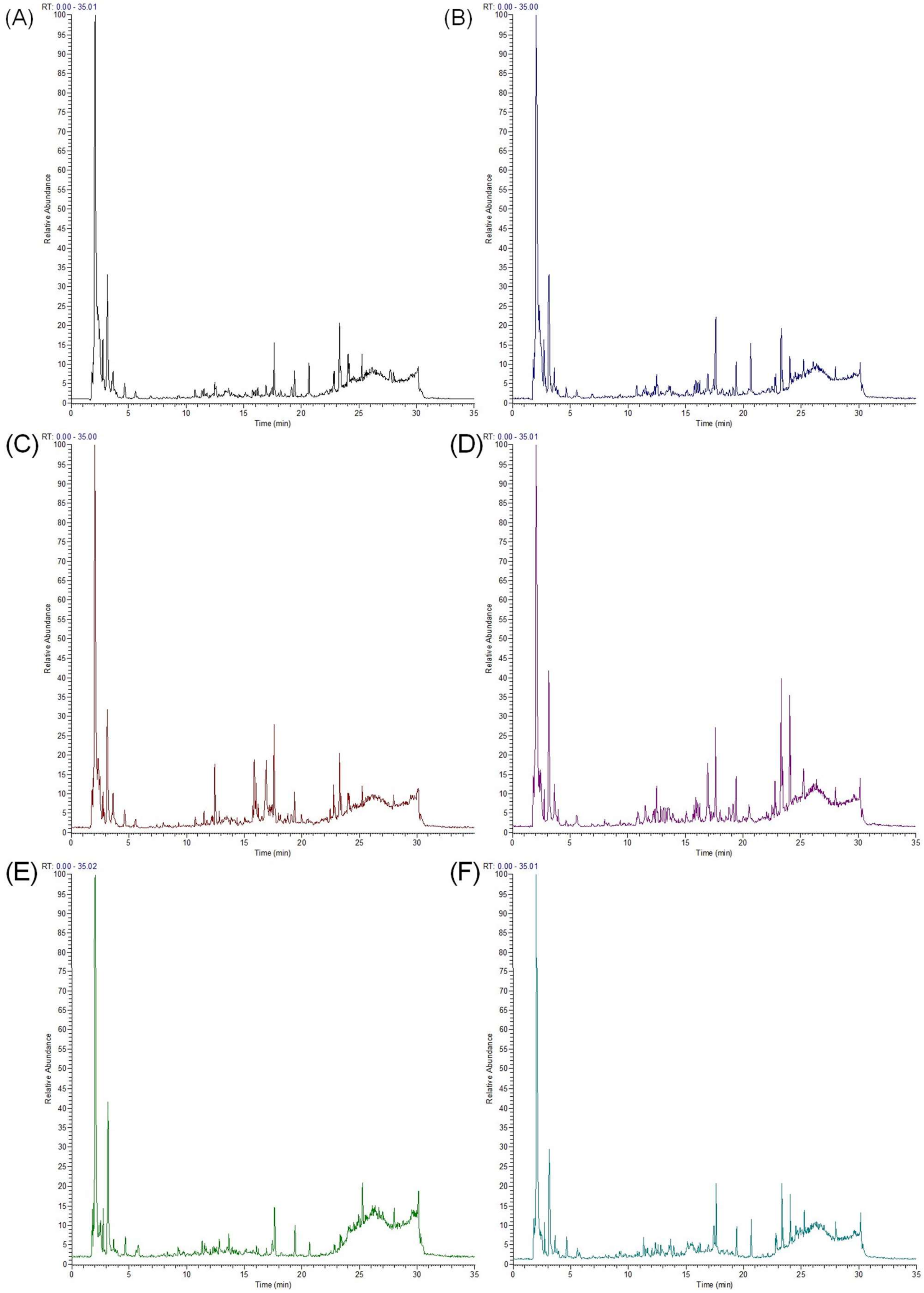

Fig. S1. Total ion chromatograms of tomato fruit (A), peel (C) and seed (E) extracts from 'Murcia' region, and tomato fruit (B), peel (D) and seed (F) extracts from 'Almería' region, obtained by LC-HRMS analysis.

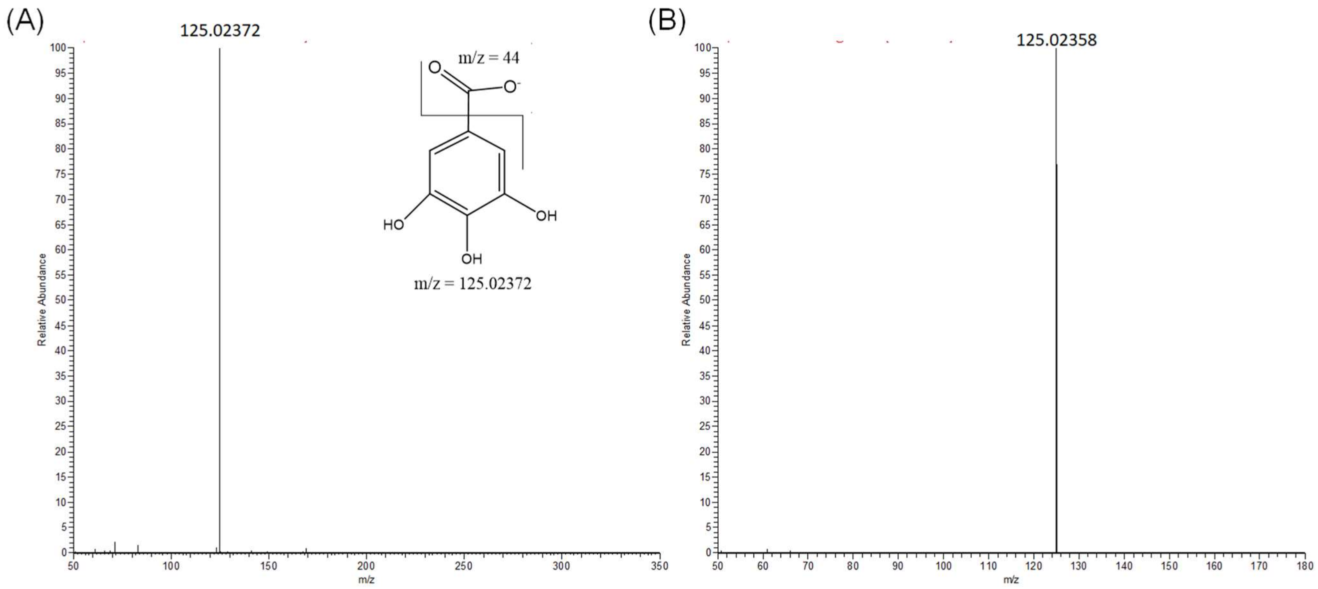

Fig. S2. HRMS/MS spectra of gallic acid from tomato and by-products extracts (A) and standard solution (B).

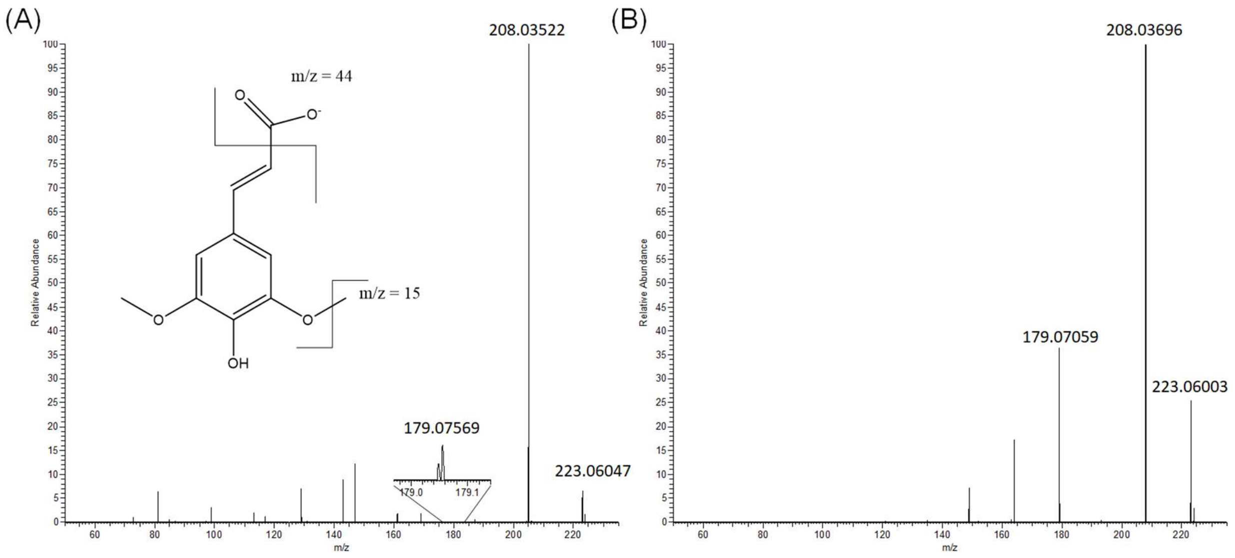

Fig. S3. HRMS/MS spectra of sinapic acid from tomato and by-products extracts (A) and standard solution (B).

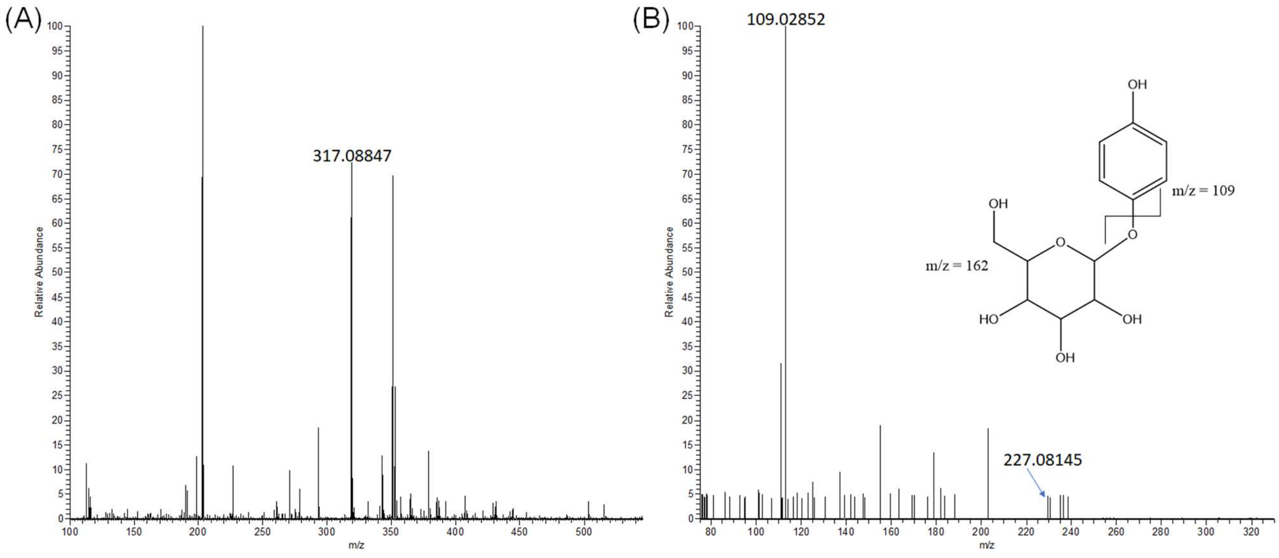

Fig. S4. HRMS spectra (A) and HRMS/MS spectra (B) of arbutin from tomato and by-products extracts.

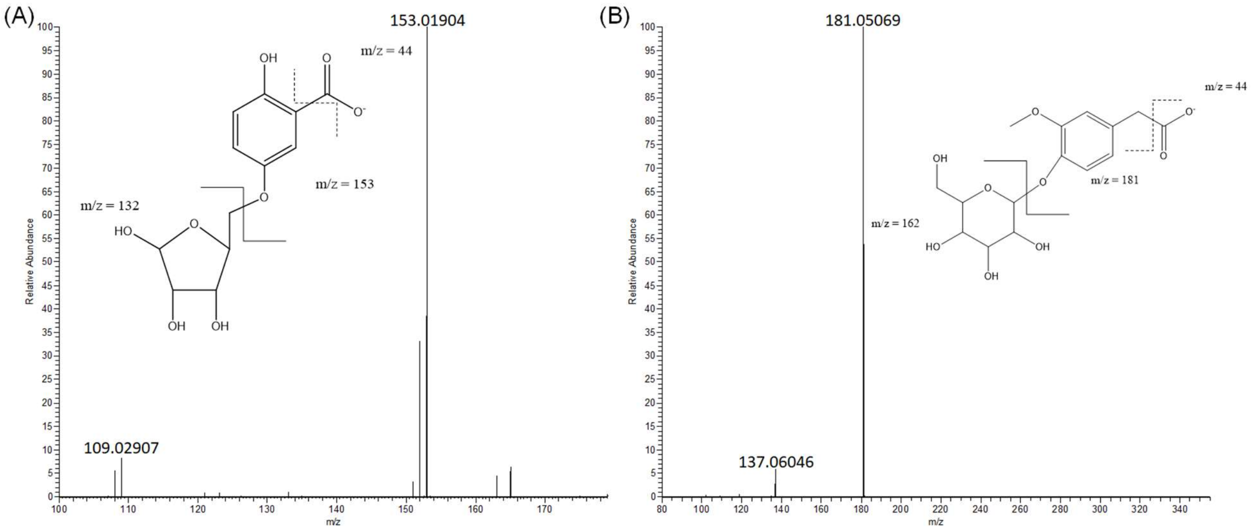

Fig. S5. HRMS/MS spectra of dihydroxybenzoic acid-O-pentoside (A) and homovanillic acid-O-hexoside (B) from tomato and by-products extracts. Dotted lines indicate a fragment for ions at *m/z* 153 and *m/z* 181 in A and B, respectively.

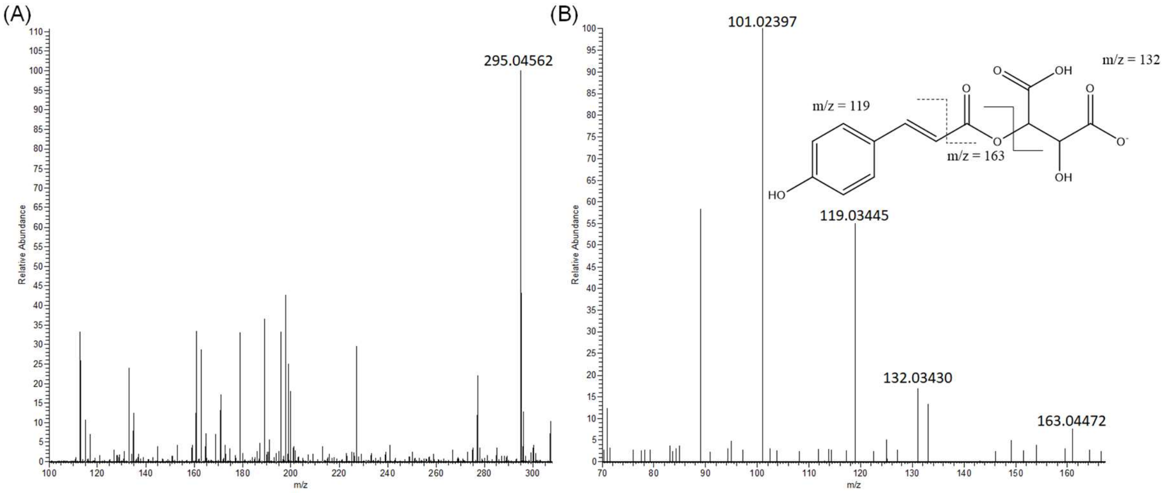

Fig. S6. HRMS spectra (A) and HRMS/MS spectra (B) of coumaroyltartaric acid from tomato and by-products extracts. Dotted line indicates a fragment for ion at *m/z* 163.

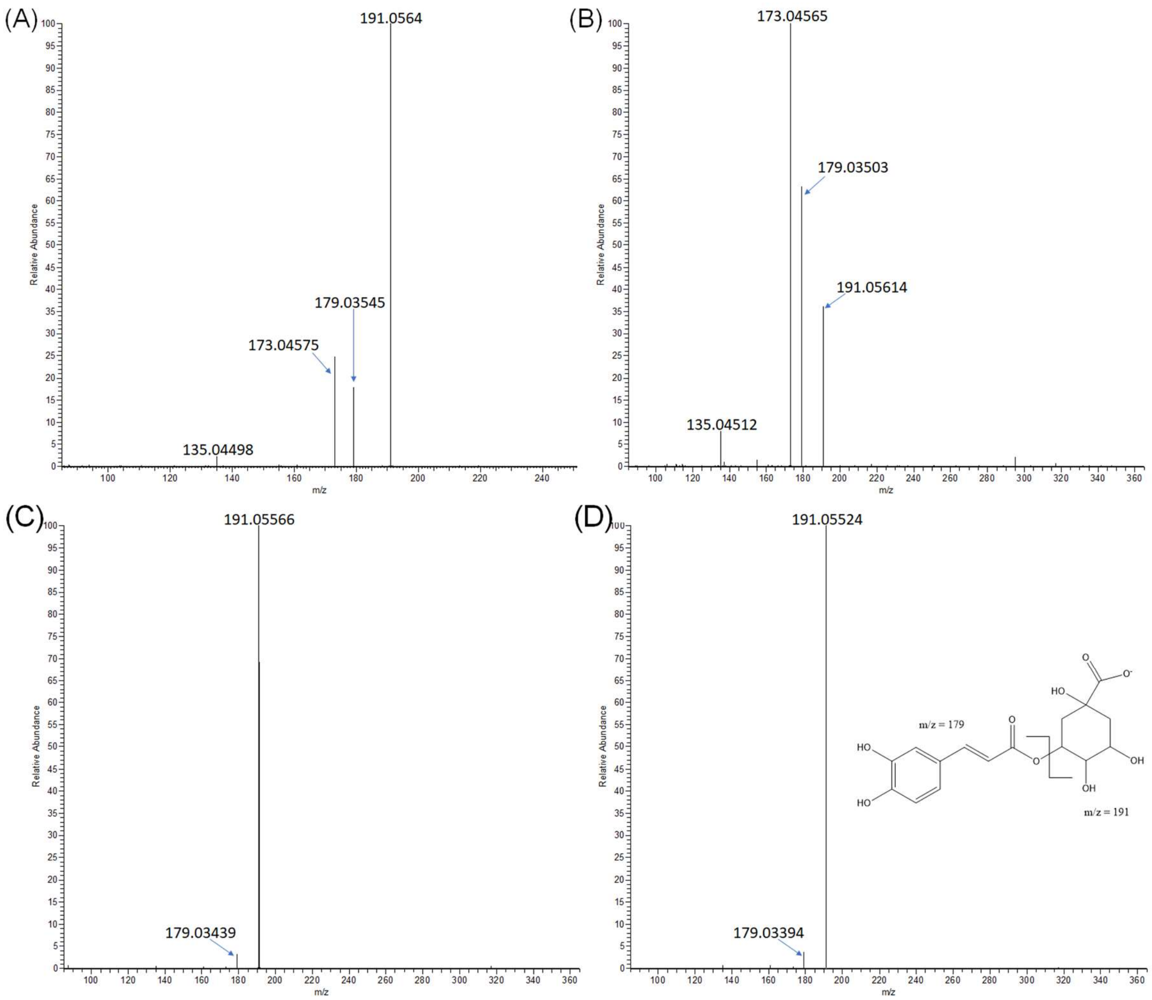

Fig. S7. HRMS/MS spectra of monocaffeoylquinic acid isomers (from A to C) from tomato and by-products extracts and chlorogenic acid standard solution (D).

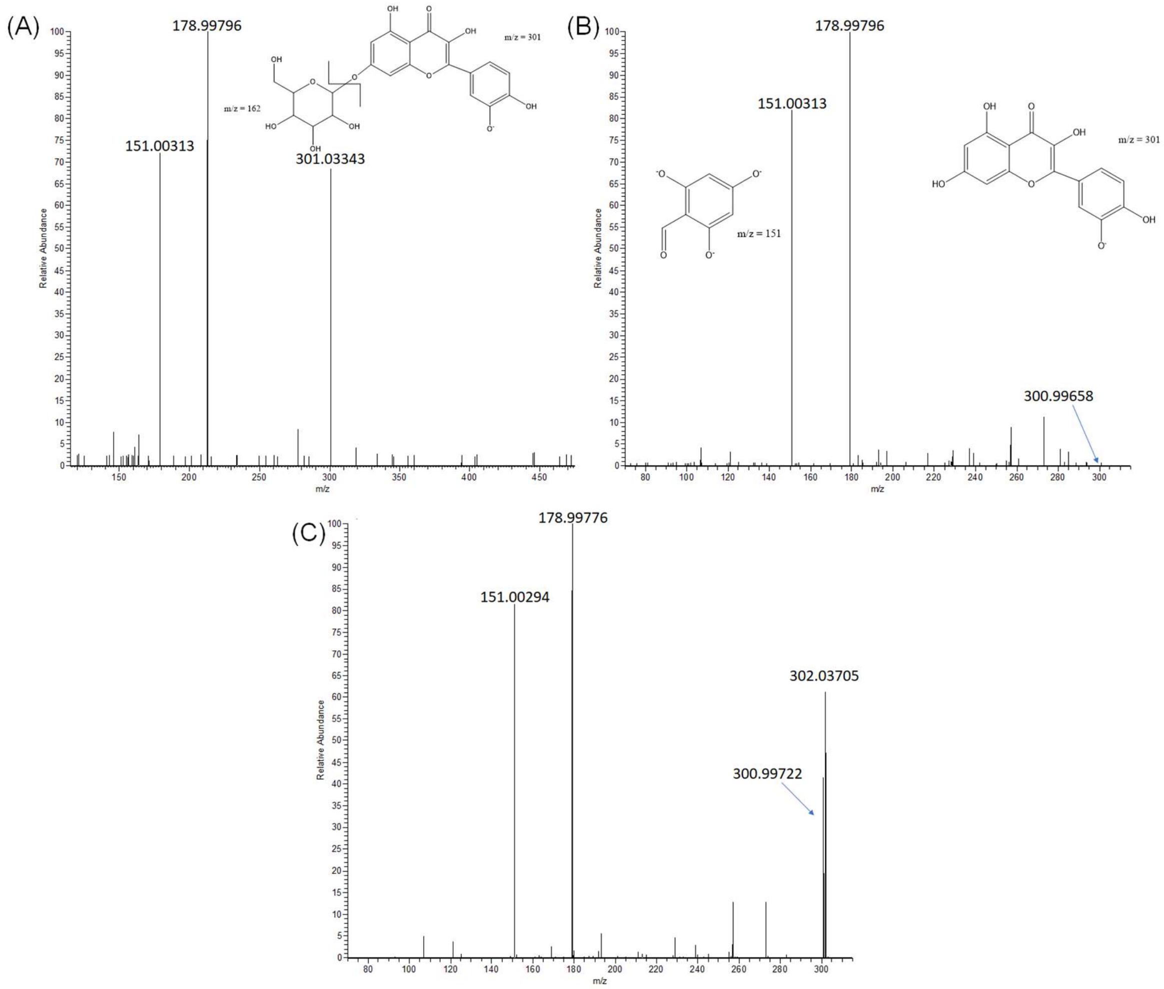

Fig. S8. HRMS/MS spectra of quecertin hexoside (A) and quercetin (B) from tomato and by-products extracts, and quercetin standard solution (C).

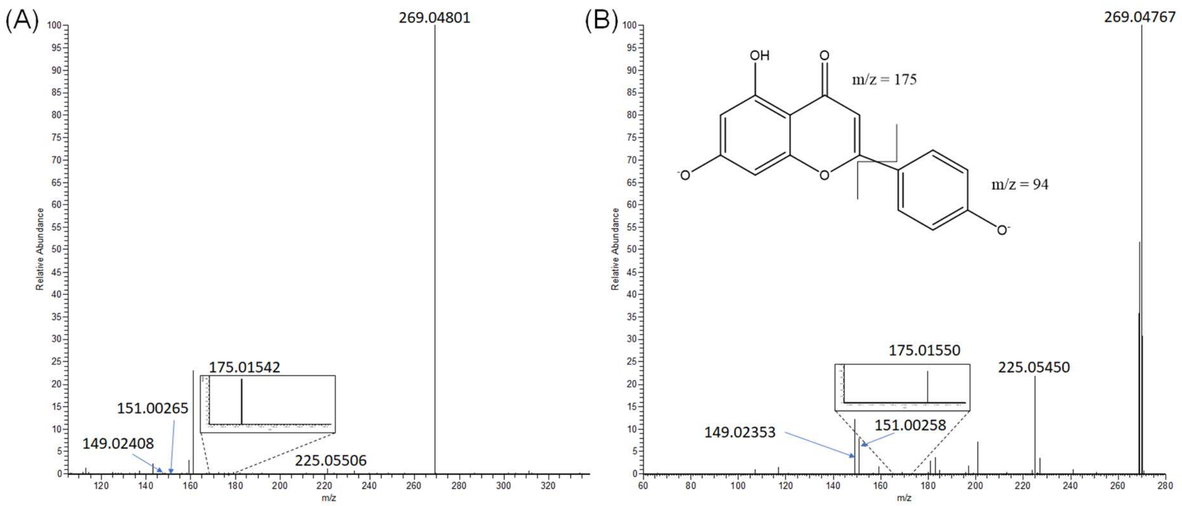

Fig. S9. HRMS/MS spectra of apigenin from tomato and by-products extracts (A) and apigenin standard solution (B).

Table S1. Classification of identified polyphenolic compounds into families.

Compounds	Phenolic family
Gallic acid	Hydroxybenzoic acids
Hydroxybenzoic acid-*O*-hexoside	Hydroxybenzoic acids
Vanillic acid	Hydroxybenzoic acids
Vanillic acid glucoside	Hydroxybenzoic acids
Dihydroxybenzoic acid isomer I	Hydroxybenzoic acids
Dihydroxybenzoic acid isomer II	Hydroxybenzoic acids
Dihydroxybenzoic acid-*O*-pentoside	Hydroxybenzoic acids
Homovanillic acid-*O*-hexoside	Hydroxybenzoic acids
4-Hydroxybenzoic acid	Hydroxybenzoic acids
Protocatechuic acid	Hydroxybenzoic acids
Homovanillic acid	Hydroxybenzoic acids
Hydroxybenzoic acid isomer	Hydroxybenzoic acids
Cinnamic acid	Hydroxycinnamic acids
Coumaroyltartaric acid isomer II	Hydroxycinnamic acids
Neochlorogenic acid	Hydroxycinnamic acids
Coumaric acid isomer I	Hydroxycinnamic acids
Dihydroferulic acid glucuronide	Hydroxycinnamic acids
Caffeic acid-*O*-hexoside	Hydroxycinnamic acids
Coumaric acid-*O*-hexoside	Hydroxycinnamic acids
Cryptochlorogenic acid	Hydroxycinnamic acids
Ferulic acid-*O*-hexoside	Hydroxycinnamic acids
Caffeic acid	Hydroxycinnamic acids
Chlorogenic acid	Hydroxycinnamic acids
Syringic acid	Hydroxycinnamic acids
Coutaric acid	Hydroxycinnamic acids
Coumaroylquinic acid	Hydroxycinnamic acids
p-Coumaric acid	Hydroxycinnamic acids
Sinapic acid	Hydroxycinnamic acids
Dicaffeoylquinic acid isomer I	Hydroxycinnamic acids
Dicaffeoylquinic acid isomer II	Hydroxycinnamic acids
Ferulic acid	Hydroxycinnamic acids
Catechin	Flavanols
Epigallocatechin	Flavanols
Eriodictyol	Flavanones
Prunin	Flavanones
Naringenin	Flavanones
Apigenin-*O*-hexoside isomer I	Flavones
Apigenin-*O*-hexoside isomer II	Flavones
Diosmin	Flavones
Diosmetin-*O*-hexoside	Flavones
Apigenin	Flavones
Myricetin	Flavonols
Rutin-*O*-pentoside	Flavonols
Kaempferol-*O*-hexoside	Flavonols
Galangin 3-[galactosyl-(1→4)-rhamnoside]	Flavonols

Table S1. continued

Rutin	Flavonols
Quercetin-*O*-hexoside isomer I	Flavonols
Kaempferol-O-rutinoside	Flavonols
Kaempferol isomer	Flavonols
Quercetin-*O*-hexoside isomer II	Flavonols
Quercetin	Flavonols
Kaempferol	Flavonols
Quercetin isomer	Flavonols
Galangin	Flavonols
Isorhamnetin	Flavonols
Phloretin-*C*-diglucoside	Chalcones
Arbutin	Others phenols
